# The first detection of *Rickettsia aeschlimannii* and *Rickettsia massiliae* in *Rhipicephalus turanicus* ticks, in northwest China

**DOI:** 10.1186/s13071-015-1242-2

**Published:** 2015-12-10

**Authors:** Qing-Qing Wei, Li-Ping Guo, An-Dong Wang, Lu-Meng Mu, Ke Zhang, Chuang-Fu Chen, Wan-Jiang Zhang, Yuan-Zhi Wang

**Affiliations:** School of Medicine, Shihezi University, Shihezi, Xinjiang Uygur Autonomous Region 832000 People’s Republic of China; School of Animal Science and Technology, Shihezi University, Shihezi, Xinjiang Uygur Autonomous Region 832000 People’s Republic of China; Pingdingshan University, Pingdingshan, Henan 467000 People’s Republic of China

**Keywords:** *Rickettsia aeschlimannii*, *Rickettsia massiliae*, *Rhipicephalus turanicus* ticks, Northwest China

## Abstract

**Background:**

*Rickettsia* spp. belonging to the spotted fever group (SFG) cause infections in humans, domestic animals and wildlife. At least five SFG rickettsial species have been reported in China, but the occurrence of *Rickettsia aeschlimannii* and *R. massiliae* in ticks has not been characterized to date.

**Findings:**

A total of 114 adult ticks were collected from sheep in Yining County, Xinjiang Uygur Autonomous Region, in northwest China. The ticks were identified from morphological and molecular characteristics. All samples were examined by polymerase chain reaction (PCR), and six genetic markers were used to determine the *Rickettsia* spp. in the ticks. The ticks collected were identified as *Rhipicephalus turanicus*. Three different lineages of *Rh. turanicus* from Yining County were discovered on phylogenetic analysis of *16S rDNA* and *cox1*. Twenty-one of the 114 samples (18.42%) were positive for rickettsial agents. Phylogenetic analysis based on six genetic sequences showed that three rickettsial species were present, namely: *R. aeschlimannii* (19.05%, 4/21), *R. massiliae* (19.05%, 4/21) and *R. sibirica* variant (61.90%, 13/21), which is clustered in the clade of *R. sibirica* subsp. *sibirica*.

**Conclusions:**

This is the first description of *R. aeschlimannii* and *R. massiliae* in China. *R. massiliae*, *R. aeschlimannii* and *R. sibirica* variant co-circulate in the region of the China-Kazakhstan border, in northwest China. Rickettsial agents in ticks of the genus *Rhipicephalus* from migrant birds, transported livestock, wildlife and human beings should be investigated further in the region of the China–Central Asian border.

**Electronic supplementary material:**

The online version of this article (doi:10.1186/s13071-015-1242-2) contains supplementary material, which is available to authorized users.

## Findings

### Background

*Rickettsia* spp. belonging to the spotted fever group (SFG) cause infections in animals and humans worldwide [1, 2]. To date, at least five validated SFG rickettsial species have been detected in ticks in China, including *R. heilongjiangii*, *R. sibirica*, *R. raoultii*, *R. slovaca* and *R. felis* [3]. Molecular evidence of the first four species was reported in northeastern and northwestern China, mainly in *Dermacentor* and *Haemaphysalis* ticks [4–6], and the last was found in *Rhipicephalus sanguineus* from Jiangsu Province [7].

Xinjiang Uygur Autonomous Region (XUAR), the largest province in China, occupies one-sixth of China, borders eight countries with a 5,600-km frontier, and there are 29 trading ports. In the present study, we assessed the occurrence of rickettsial agents in *Rh. turanicus* ticks in Yining County, the location of Yining Port, which is adjacent to Kazakhstan.

## Methods

### Tick sampling and identification

A total of 114 ticks were collected from sheep in Yining County (928 m above sea level, at 44°003681′N 81°558182′E). All of the ticks were identified morphologically according to previous reports, and 23 representative ticks underwent molecular analysis based on partial mitochondrial (*16S rDNA* and *cox1*) gene sequences [8].

### Ethical approval

This study was approved by the Animal Ethics Committee of Shihezi University (Approral No. AECSU2014-6).

### PCR amplification and sequence analysis

For genetic detection of *Rickettsia* spp., the genomic DNA of all the ticks was extracted from individual specimens using the TIANamp Genomic DNA Kit (Tiangen, Beijing, China). All samples were examined by polymerase chain reaction (PCR), and six genetic markers [434-, 1332-, 1060-, 488-, 491-, and 812-bp products of the genes encoding the 17 kilodalton antigen (*17-kDa*), 16S rRNA (*rrs*), citrate synthase (*gltA*), surface cell antigen 1 (*sca1*), and outer membrane proteins A and B (*ompA* and *ompB*)] were amplified using previously described primers [3]. The amplication products were purified using the TIANgel Midi Purification Kit (TIANGEN, Beijing, China) and then cloned into the pGEM-T Easy vector and subjected to sequencing. A phylogenetic tree was constructed using the maximum likelihood and neighbor-joining algorithms with MEGA 6.0 software [9].

## Results

The ticks were identified morphologically as *Rh. turanicus*. Sequencing data from the 23 representative ticks indicated three different lineages of *Rh. turanicus* from Yining County on the basis of phylogenetic analysis of *16S rDNA* and *cox1* (shown in Additional file 1). Six nucleotide sequences from our study have been deposited in the GenBank database (*16S rDNA*: KF547984, KF547987, and KF547989; *cox1*: KF188136–KF188138).

**Fig. 1:**
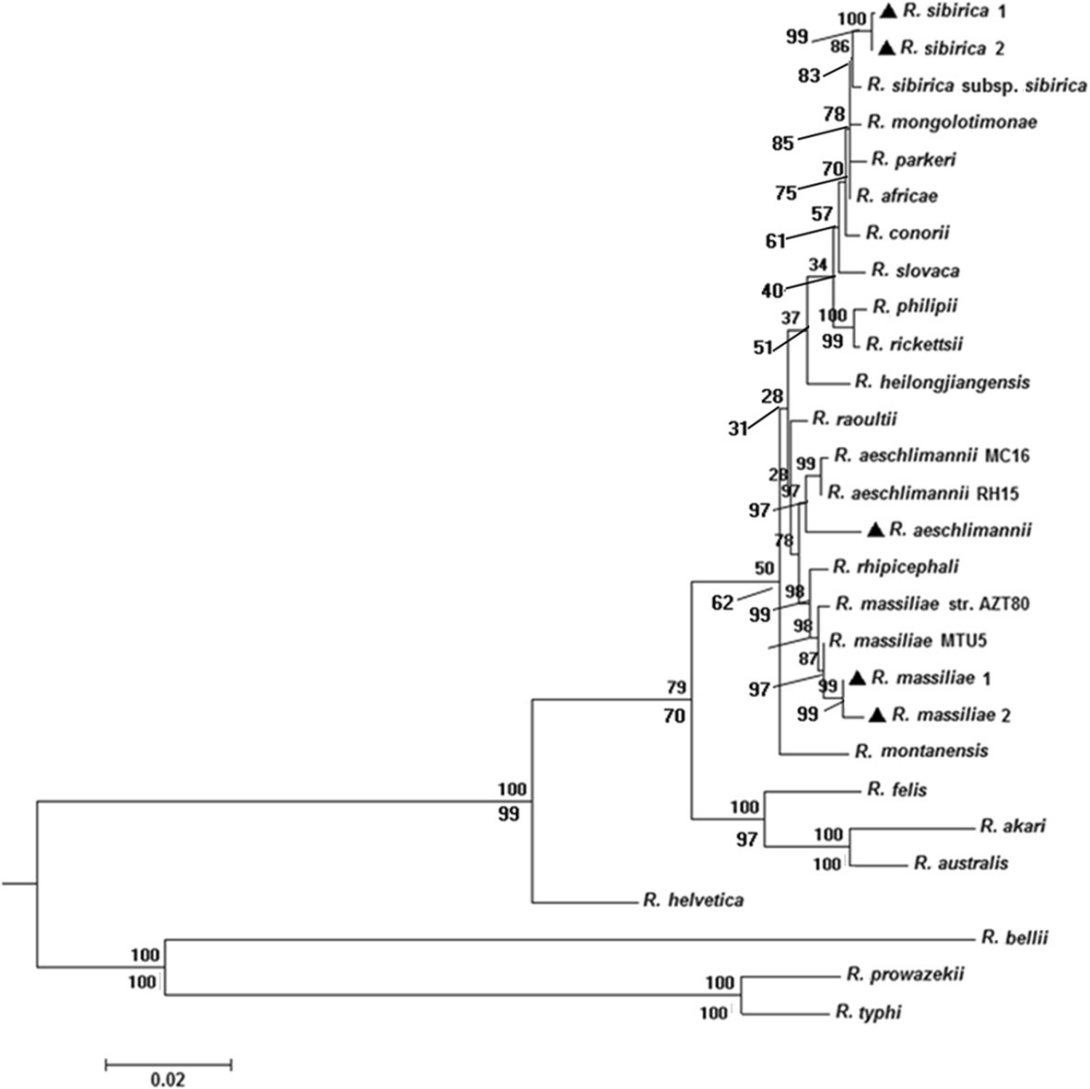
Phylogenetic tree of the *17-kDa-ompA-gltA-rrs-sca1-ompB* concatenated sequence of rickettsial agents in *Rhipicephalus turanicus* (). The tree was constructed on the basis of maximum likelihood (ML; bootstrap replicates: 500) and neighbor-joining (NJ; bootstrap replicates: 500) analyses of concatenated sequence data for six genes (*17-kDa*, *ompA*, *gltA*, *rrs*, *sca1*, *ompB*) using MEGA6. The scale bar represents the inferred substitutions per nucleotide site. The relative support for clades in the tree produced from the ML and NJ analyses are indicated above and below branches, respectively

Twenty-one of the 114 samples (18.42%) were positive by PCR for products of six rickettsial genetic markers. Out of the 21 positive samples, four were confirmed as *R. aeschlimannii*, four were identified as *R. massiliae*, and the remaining thirteen were *R. sibirica* variant based on phylogenetic tree of the representative makers (*ompA* gene and *gltA* gene) and the *17-kDa-ompA-gltA-rrs-sca1-ompB* concatenated sequence (shown in Additional file 2; Fig. 1). There were no differences in the DNA sequences of six responding genetic markers for *R. aeschlimannii,* with sequence similarities of 99.74% (1,169bp/1,172bp), 100% (1,048bp/1,048bp), 98.49% (458bp/465bp), 98.77% (722bp/731bp) and 99.33% (593bp/597bp) for the *rrs*, *gltA*, *ompA*, *ompB* and *sca1* genes, respectively, and 99.19% (366 bp/369bp) to *R. raoultii* strain Alashankou-99 for the *17k-Da* gene (KT261761). Except the *sca1* gene, which has two different sequences with sequence similarities of 99.13% (573bp/578bp) and 99.48% (576bp/579bp) to *R. massiliae* MTU5 (CP000683), and the *ompB* gene, which has two different sequences with sequence similarities of 100% (765bp/765bp) and 98.56% (754bp/765bp) to *R. massiliae* MTU5 (CP000683), the DNA sequences of four genetic markers for *R. massiliae* were the same, with sequence similarities of 100% (383bp/383bp), 100% (1,162bp/1,162bp), 99.90% (1,022bp/1,023bp), 100% (434bp/434bp) for the *17k-Da, rrs*, *gltA*, *ompA* genes, respectively. However, for the *R. sibirica* variant, except the *gltA* gene, which has two different sequences with sequence similarities of 99.54% (1,075bp/1,080bp) and 99.63% (1,076bp/1,080bp) to *R. sibirica* subsp. *sibirica* (KM28871), respectively, the sequences of the other five responding genetic markers have different levels of divergences, with sequence similarities of 100% (385bp/385bp) to *R. raoultii* strain Alashankou-131(KT261760) for the *17k-Da* gene, 99.82% (1,121bp/1,123bp) to *R. raoultii* isolate BL029-2 (KJ410261) for the *rrs* gene, 99.58% (469bp/471bp) to *Rickettsia* sp. Tselentii (EU194445) for the *ompA* gene, 99.48% (772bp/776bp) to *R. parkeri* str. Portsmouth (CP003341) for the *ompB* gene and 99.34 (598/602) to *R. africae* ESF-5 (CP001612) for the *sca1* gene. The similarities and divergences of the sequences in this study are shown in Additional file 3: Table S1. All the sequences obtained from our study have been deposited in the GenBank database [*17 kDa*: KT318742, KT588057, KT588065; *rrs*: KT318741, KT588056, KT588064; *gltA*: KT318743, KT588058, KT588066, KT588070; *sca1*: KT318746, KT588061, KT588063, KT588069; *ompA*: KT318744, KT588059, KT588067; *ompB*: KT318745, KT588060, KT588062, KT588068].

## Discussion

*R. massiliae*, *R. rhipicephali* and *R. aeschlimannii* are grouped phylogenetically into a clade in the family *Rickettsiaceae* [10]. *R. massiliae* was first isolated in 1990 from a *Rh. turanicus* tick in an area near Marseille, France [11]. Since then, this pathogen has been identified from other *Rhipicephalus* ticks in regions of Europe, North and Central Africa, and the United States [12]. Furthermore, cases showed that it can cause human infection. *R. aeschlimannii* was first described from *Hyalomma marginatum* in Morocco in 1997 [13]. The presence of *R. aeschlimannii* has been demonstrated in *Hyalomma* ticks from Europe (e.g. France, Croatia, Spain, Italy), Asia (e.g. Israel, Turkey) and Africa (e.g. Mali, Algeria, Egypt) [14–16] and from *Haemaphysalis* ticks in Spain and Kazakhstan [17]. Furthermore, *Ixodes ricinus*, *H. punctata*, *Rh. bursa*, and *Rh. sanguineus* isolated from human Spanish patients were shown to contain DNA from *R. aeschlimannii* [14], and there is a report of *R. aeschlimannii* from *Rh. turanicus* infecting a man in Greece in 2013 [18]. In this study, we report the first molecular evidence that *R. aeschlimannii* and *R. massiliae* are present in *Rh. turanicus* from sheep in the region of the China-Kazakhstan border, in the northwest of China.

To date, *R. sibirica* is known to contain two subspecies [19], *R. sibirica* subsp. *sibirica* and *R. sibirica* subsp. *mongolotimonae*. The former was first isolated in Russia but it has subsequently been found in northern China [5]. In contrast, *R. sibirica* subsp. *mongolotimonae* was first isolated in Inner Mongolia and then found in Europe and Africa [20, 21]. Here, the *R. sibirica* variant found in the region of the China–Kazakhstan border appeared divergent in the *ompA*, *ompB* and *sca1*, used to differentiate *Rickettsia* species, although it was closest to *R. sibirica* subsp. *sibirica*, on the basis of the *gltA* gene and the phylogenetic tree of the *17-kDa-ompA-gltA-rrs-sca1-ompB* concatenated sequence. Further genomic analysis should be carried out to confirm the classification of the *R. sibirica* variant found in this study.

The *Rh. turanicus* tick is widely distributed throughout the Mediterranean subregion, Africa, and Asia, including China, especially in XUAR [22], and it has been implicated as a vector of several human and veterinary pathogens, including *Rickettsia* spp. [18]. Here, *R. massiliae*, *R. aeschlimannii* and *R. sibirica* variant were found in the same area, Yining County, which suggests that several SFG *Rickettsia* spp. co-circulate in *Rh. turanicus* as a potential vector near the China-Kazakhstan border.

In 2004, Shpynov *et al* detected *R. aeschlimannii* in the Alma-Ata region, east of Kazakhstan [17]. Here we found that *Rh. turanicus* in the region of the China-Kazakhstan border showed genetic divergence in the loci of *16S rDNA* and *cox1*, which indicates that these ticks collected from sheep may come from different lineages. At present, it is unknown whether these ticks are imported from the Chinese hinterland or abroad through migrant birds, or with internationally transported livestock. This topic needs to be further investigated.

## Conclusions

This is the first report of the molecular analysis of *R. aeschlimannii* and *R. massiliae* in China. The findings of the study suggest that *R. massiliae*, *R. aeschlimannii* and *R. sibirica* variant co-circulate in *Rh. turanicus* in the region of the China–Kazakhstan border, in northwest China. The origin of the *Rhipicephalus genus* (such as migrant birds, transported livestock, wildlife and human beings) and the epidemiology of tick-borne pathogens should be further investigated in the region of the China–Central Asian border.

## Additional files

Additional file 1: **The photo of** ***Rhipicephalus turanicus*** **and Phylogenetic tree of** ***Rhipicephalus turanicus 16S rDNA*** **and** ***CO1*** **gene.** (DOC 198 kb) Additional file 2: **The single gene Phylogenetic tree of** ***Rickettsia*** **spp.** (DOC 863 kb) Additional file 3:
**Closest relative sequences to the partial** ***17-kDa***, ***16S***, ***gltA***, ***ompA***, ***ompB*** **and** ***sca1*** **genes, sequences of the** ***Rickettsia aeschlima*** **nnii (Table S1A),** ***Rickettsia massiliae*** **(Table S1B) and** ***Rickettsia sibirica*** **(Table S1C) detected in the** ***Rhipicephalus turanicus*** **ticks, Northwest of China.** (DOCX 22 kb)
