# Supplementary figures and images for: The first detection of Rickettsia aeschlimannii and Rickettsia massiliae in Rhipicephalus turanicus ticks, in northwest China
Source: Parasit Vectors. 2015 Dec 10;8:631. doi: 10.1186/s13071-015-1242-2 (PMC4675064; doi:10.1186/s13071-015-1242-2)

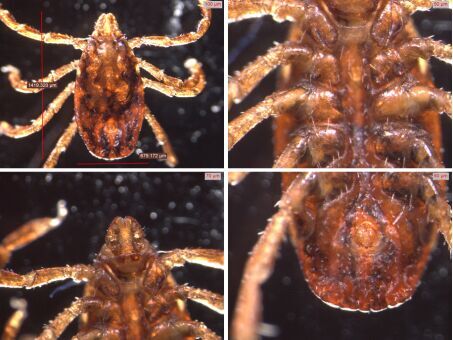


*Rhipicephalus turanicus*


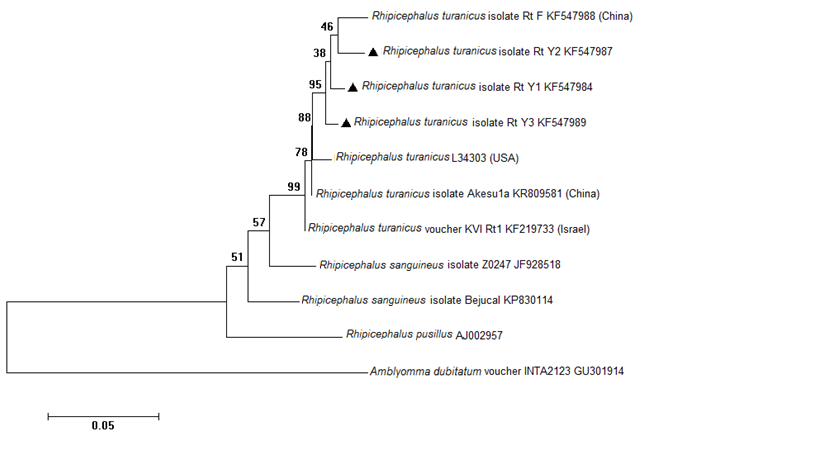


*Rhipicephalus turanicus* 16S rDNA -ML


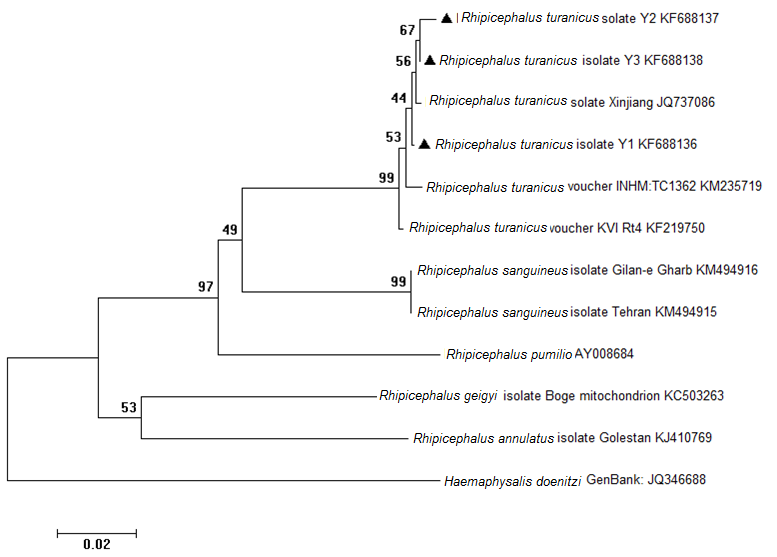


*Rhipicephalus turanicus* CO1- NJ

Supplement: Additional file 1: — The photo of Rhipicephalus turanicus and Phylogenetic tree of Rhipicephalus turanicus 16S rDNA and CO1 gene. (DOC 198 kb) [file 13071_2015_1242_MOESM1_ESM.doc]

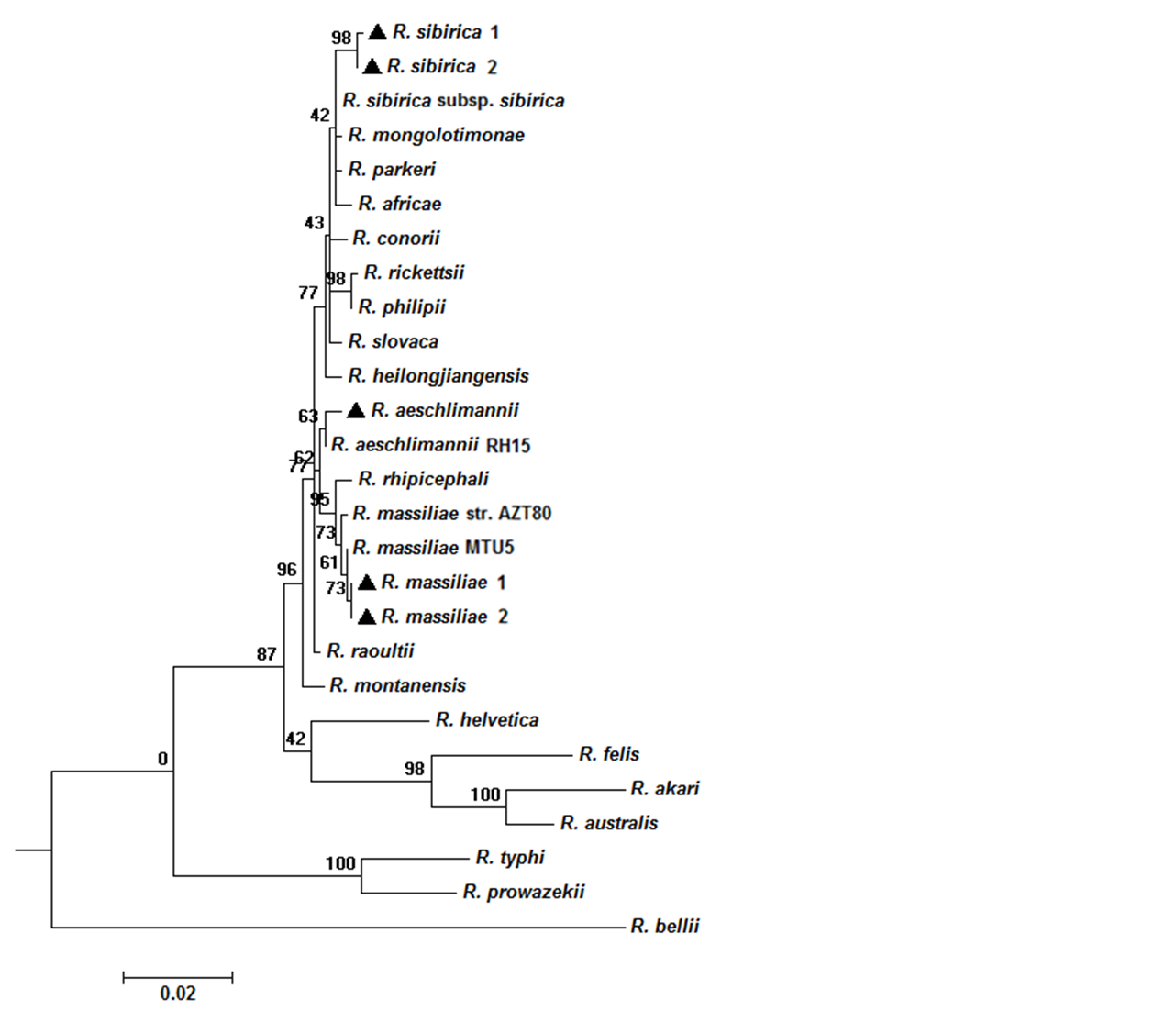


gltA-ML


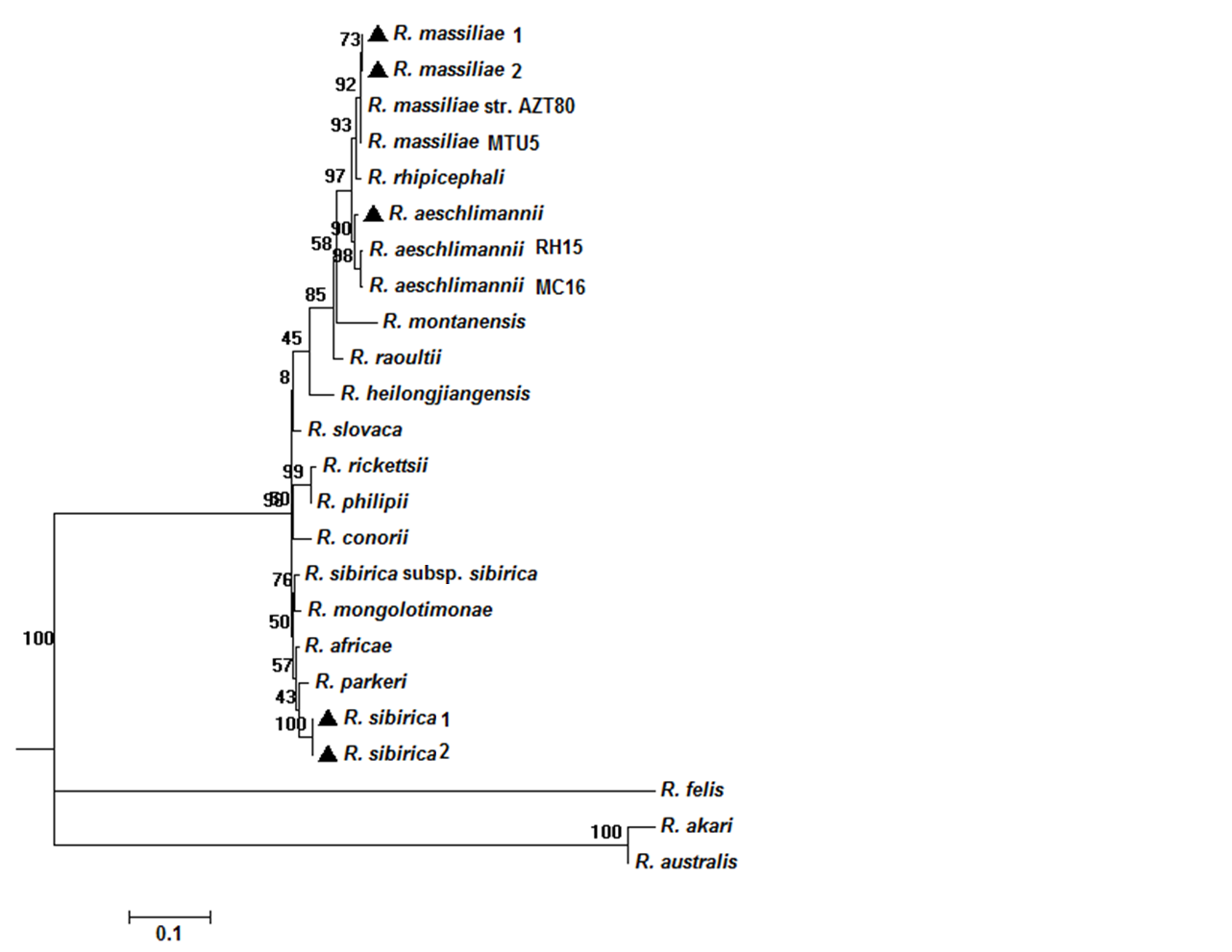


ompA-ML


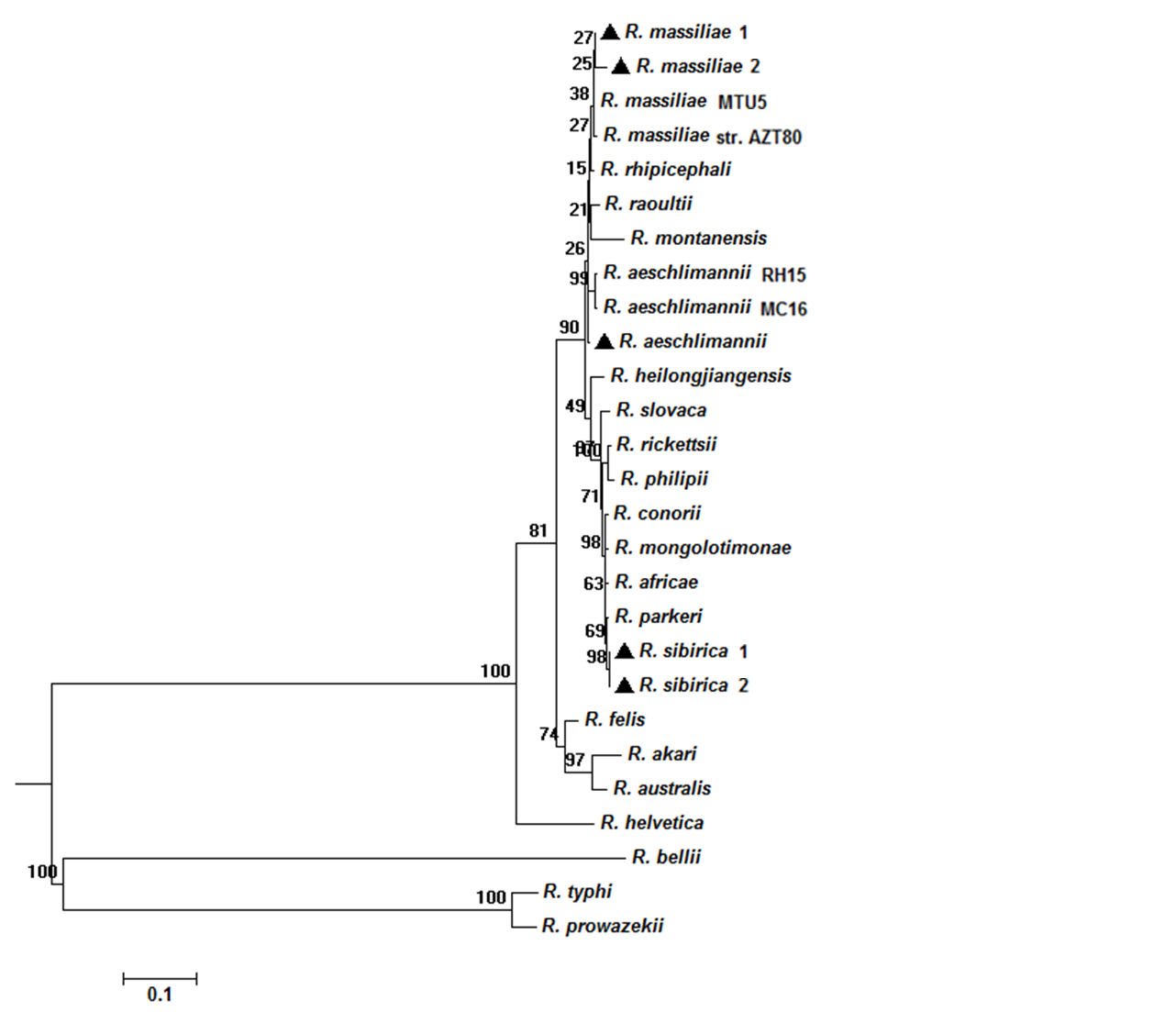


ompB-ML


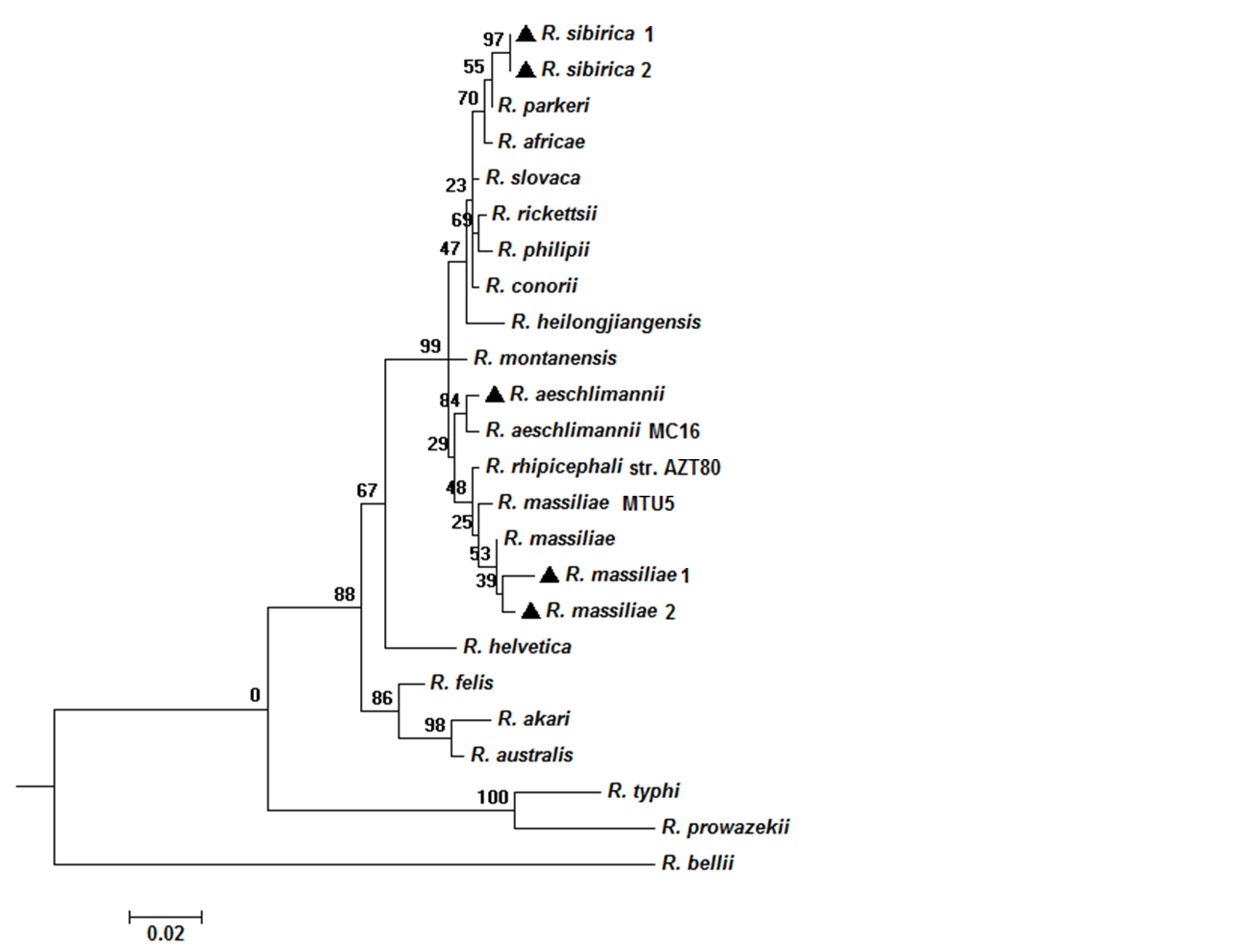


sca1-ML

Supplement: Additional file 2: — The single gene Phylogenetic tree of Rickettsia spp. (DOC 863 kb) [file 13071_2015_1242_MOESM2_ESM.doc]
