# Supplementary material for: The first detection of Rickettsia aeschlimannii and Rickettsia massiliae in Rhipicephalus turanicus ticks, in northwest China
Source: Parasit Vectors. 2015 Dec 10;8:631. doi: 10.1186/s13071-015-1242-2 (PMC4675064; doi:10.1186/s13071-015-1242-2)
Supplement: Additional file 3: — Closest relative sequences to the partial 17-kDa, 16S, gltA, ompA, ompB and sca1 genes, sequences of the Rickettsia aeschlima nnii (Table S1A), Rickettsia massiliae (Table S1B) and Rickettsia sibirica (Table S1C) detected in the Rhipicephalus turanicus ticks, Northwest of China. (DOCX 22 kb) [file 13071_2015_1242_MOESM3_ESM.docx]

**Appendix Table 1**

Closest relative sequences to the partial *17-kDa*, *16S*, *gltA*, *ompA*, *ompB* and *sca1* genes, sequences of the *Rickettsia* *aeschlima*nnii (Appendix Table 1A), *Rickettsia massiliae* (Appendix Table 1B) and *Rickettsia sibirica* (Appendix Table 1C) detected in the *Rhipicephalus turanicus* ticks, Northwest of China.

| Gene | *Rickettsia* (GenBank accession No.) | % Sequence similarity(bp) |
| --- | --- | --- |
| **Appendix Table 1A** | | |
| *17-kDa* (KT318742) | *Rickettsia raoultii* strain Alashankou-99 (KT261761) | 99.19 (366/369) |
|  | *Rickettsia raoultii* strain Alashankou-105 (KT261759) | 99.19 (366/369) |
|  | *Rickettsia raoultii* strain Alashankou-148 (KT261758) | 99.19 (366/369) |
|  | *Rickettsia raoultii* (KR608784 ) | 99.19 (366/369) |
|  | *Rickettsia aeschlimannii* clone IE16 (DQ379977) | 98.91 (362/366) |
| *16S (*KT318741) | *Rickettsia aeschlimannii* strain RH15  (HM050274) | 99.74 (1169/1172) |
|  | *Rickettsia aeschlimannii* strain Mc16 (NR_026042) | 99.74 (1169/1172) |
|  | *Rickettsia raoultii* isolate TC250-11 (KJ410259) | 99.74 (1168/1171) |
| *gltA* (KT318743) | *Rickettsia aeschlimannii* (AY259084) | 100 (1048/1048) |
|  | *Rickettsia aeschlimannii* strain RH15 (HM050285) | 99.71 (1045/1048) |
|  | Rickettsia sp. MC16 citrate synthase (RSU59722) | 99.71(1045/1048) |
|  | *Rickettsia aeschlimannii* (HQ335153) | 99.71 (1044/1048) |
| *ompA* (KT318744) | *Rickettsia aeschlimannii* isolate BB-46/Ank-H.marg (KF791252) | 98.49 (458/465) |
|  | *Rickettsia aeschlimannii* isolate BB-35/Camli H.marg (KF791251) | 98.49 (458/465) |
|  | *Rickettsia aeschlimannii* isolate BB-17/Ank-H.marg (KF791250) | 98.49 (458/465) |
|  | *Rickettsia aeschlimannii* isolate BB-13/Bala H.marg (KF791247) | 98.49 (458/465) |
| *ompB* (KT318745) | *Rickettsia aeschlimannii* strain RH15 ( HM050278) | 98.77 (722/731) |
|  | *Rickettsia rhipicephali* str. 3-7-female6 CWPP (CP003342) | 98.63 (721/731) |
| *sca1* (KT318746) | *Rickettsia aeschlimannii* strain MC16 (AY355353) | 99.33 (593/597) |
| **Appendix Table 1B** | | |
| *17-kDa* (KT588057) | *Rickettsia massiliae* MTU5 (CP000683) | 100 (383/383) |
| *16S* (KT588056) | *Rickettsia massiliae* MTU5( NR_074486) | 100 (1162/1162) |
|  | *Rickettsia massiliae* MTU5 (CP000683) | 100 (1162/1162) |
|  | *Rickettsia massiliae* strain Mtu1 (NR_025919) | 100 (1162/1162) |
| *gltA* (KT588058) | *Rickettsia massiliae* MTU5( CP000683) | 99.90 (1022/1023) |
|  | *Rickettsia massiliae* Mtu 1( RMU59719) | 99.90 (1022/1023) |
|  | *Rickettsia massiliae* strain GL041 (JN043507) | 99.80 (1021/1023) |
|  | *Rickettsia massiliae* str. AZT80( CP003319 ) | 99.70 (1020/1023) |
| *ompA* (KT588059) | *Rickettsia massiliae* strain 56m( KJ663747 ) | 100 (434/434) |
|  | *Rickettsia massiliae* MTU5(CP000683) | 100 (434/434) |
| *ompB* (KT588062) | *Rickettsia massiliae* MTU5(CP000683) | 100 (765/765) |
|  | *Rickettsia massiliae* (AF123714) | 100 (765/765) |
|  | *Rickettsia massiliae* strain AZT80 (DQ503428) | 98.96 (760/768) |
| *ompB* (KT588060) | *Rickettsia massiliae* MTU5(CP000683) | 98.56 (754/765) |
|  | *Rickettsia massiliae* (AF123714) | 98.56 (754/765) |
|  | *Rickettsia massiliae* strain AZT80 (DQ503428) | 97.65 (750/768) |
| *sca1* (KT588061) | *Rickettsia massiliae* MTU5 (CP000683) | 99.13 (573/578) |
|  | *Rickettsia massiliae* strain Mtu1(AY355364) | 99.13 (573/578) |
| *sca1* (KT588063) | *Rickettsia massiliae* MTU5 (CP000683) | 99.48 (576/579) |
|  | *Rickettsia massiliae* strain Mtu1(AY355364) | 99.48 (576/579) |
| **Appendix Table 1C** | | |
| *17-kDa* (KT588065) | *Rickettsia raoultii* strain Alashankou-131﹙KT261760﹚ | 100 (385/385) |
|  | *Rickettsia rickettsii* str. Morgan﹙CP006010﹚ | 99.74 (385/386) |
|  | *Rickettsia parkeri* str. Portsmouth﹙CP003341﹚ | 99.74 (385/386) |
|  | *Rickettsia philipii* str. 364D﹙CP003308﹚ | 99.74 (385/386) |
| *16S* (KT588064) | *Rickettsia raoultii* isolate BL029-2 ﹙KJ410261﹚ | 99.82 (1121/1123) |
|  | *Rickettsia conorii* strain Malish 7 ﹙NR_074480﹚ | 99.82 (1121/1123) |
|  | *Rickettsia slovaca* isolate TC250-17﹙KJ410262 ﹚ | 99.73 (1120/1123) |
|  | *Rickettsia sibirica* strain RH05 ﹙HM050271﹚ | 99.73 (1120/1123 ) |
| *gltA* (KT588066) | *Rickettsia sibirica* subsp. sibirica﹙KM28871﹚ | 99.54 (1075/1080) |
|  | *Rickettsia sibirica* 246 ﹙RSU59734﹚ | 99.54 (1075/1080) |
|  | *Rickettsia* sp. BJ-90﹙AF178035﹚ | 99.54 (1075/1080) |
|  | *Rickettsia parkeri* str. Portsmouth﹙CP003341﹚ | 99.44 (1074/1080) |
| *gltA* (KT588070) | *Rickettsia sibirica* subsp. sibirica﹙KM28871﹚ | 99.63 (1076/1080) |
|  | *Rickettsia sibirica* 246 ﹙RSU59734﹚ | 99.63 (1076/1080) |
|  | *Rickettsia* sp. BJ-90﹙AF178035﹚ | 99.63 (1076/1080) |
|  | *Rickettsia parkeri* str. Portsmouth﹙CP003341﹚ | 99.54 (1075/1080) |
| *ompA* (KT588067) | *Rickettsia* sp. Tselentii﹙EU194445﹚ | 99.58 (469/471) |
|  | *Candidatus* Rickettsia barbariae ﹙EU272186﹚ | 99.36 (468/471) |
|  | *Rickettsia africae* (JQ691730) | 97.26 (461/474) |
|  | *Rickettsia parkeri* ﹙KJ158741﹚ | 97.05 (460/474) |
| *ompB* (KT588068) | *Rickettsia parkeri* str. Portsmouth﹙CP003341﹚ | 99.48 (772/776) |
|  | *Rickettsia sibirica* strain RH05﹙HM050273﹚ | 99.10 (769/776) |
|  | *Rickettsia africae* ESF-5﹙CP001612﹚ | 99.10 (769/776) |
|  | *Rickettsia mongolotimonae* ﹙DQ097083﹚ | 99.10 (769/776) |
| *sca1* (KT588069) | *Rickettsia africae* ESF-5﹙CP001612﹚ | 99.34 (598/602) |
|  | *Rickettsia parkeri* str. Portsmouth﹙CP003341﹚ | 99.00 (596/602) |
|  | *Rickettsia conorii* ﹙AY502117 ﹚ | 98.84 (595/602) |
|  | *Rickettsia sibirica* ﹙ AY355356 ﹚ | 98.67 (594/602) |
